# Unlocking extracellular mitochondria from bench to clinical application in stroke

**DOI:** 10.1093/stcltm/szaf060

**Published:** 2025-11-14

**Authors:** Gen Hamanaka, Dong-Bin Back, Ji-Hyun Park, Shin Ishikane, Kazuhide Hayakawa

**Affiliations:** Neuroprotection Research Laboratories, Departments of Radiology and Neurology, Massachusetts General Hospital and Harvard Medical School, Charlestown, MA 02129, United States; Neuroprotection Research Laboratories, Departments of Radiology and Neurology, Massachusetts General Hospital and Harvard Medical School, Charlestown, MA 02129, United States; Neuroprotection Research Laboratories, Departments of Radiology and Neurology, Massachusetts General Hospital and Harvard Medical School, Charlestown, MA 02129, United States; Neuroprotection Research Laboratories, Departments of Radiology and Neurology, Massachusetts General Hospital and Harvard Medical School, Charlestown, MA 02129, United States; Neuroprotection Research Laboratories, Departments of Radiology and Neurology, Massachusetts General Hospital and Harvard Medical School, Charlestown, MA 02129, United States

**Keywords:** biomarkers, central nervous system, extracellular mitochondria, mitochondria transplantation, mitochondrial modification, stroke

## Abstract

Within the central nervous system (CNS), mitochondria serve as vital energy sources for neurons, glial cells, and vascular functions, maintaining intracellular metabolic balance. Recent studies involving cellular models, rodents, and humans reveal that metabolically active mitochondria can be released into the extracellular space, playing roles in intercellular communication within the CNS. When taken up by neurons, these extracellular mitochondria may provide neuroprotective effects. Conversely, damaged mitochondria and their released components during severe tissue injury or inflammation can contribute to neurodegenerative processes. Thus, mitochondria secreted under pathological conditions in the CNS hold promise as biomarkers indicative of recovery. Additionally, transplantation of external mitochondria shows potential as a therapeutic approach for various CNS disorders. This mini review focuses on recent advances in the transfer of mitochondria between cells, the use of extracellular mitochondria as biomarkers, and the prospects of mitochondria transplantation from experimental research to clinical application, particularly in diseases like stroke.

Significance Statement Mitochondria are central to neuronal, glial, and vascular metabolism in the CNS. Emerging evidence shows that functional mitochondria can be released into the extracellular space, mediating intercellular communication, and supporting neuronal recovery. In contrast, damaged mitochondria released during injury may amplify inflammation and neurodegeneration. These dual roles highlight extracellular mitochondria as potential biomarkers of injury and repair. Moreover, mitochondrial transplantation has shown therapeutic promise for CNS diseases, including stroke, bridging experimental insights toward clinical translation.

## Introduction

Mitochondria serve as the primary energy generators within cells and are crucial for sustaining cellular functions in mammals.^1^ The brain, being a highly metabolic organ, relies heavily on mitochondria to maintain nearly all facets of cellular homeostasis. These organelles produce most of the cell’s adenosine triphosphate (ATP),^2^^,^^3^ facilitate fatty acid biosynthesis,^4^ regulate calcium storage within cells,^5^^,^^6^ and provide a platform for signaling networks that influence cell survival, immune responses, and autophagy processes.^7^ In the setting of central nervous system (CNS) injury or disease, the buildup of mitochondrial reactive oxygen species (ROS), inflammasomes, and disrupted mitochondrial membrane permeability contribute to cell death and neuroinflammation.^8^^,^^9^ Consequently, targeting mitochondrial dysfunction has emerged as a key therapeutic approach for various CNS conditions, including stroke,^10^ hemorrhagic stroke,^11^ spinal cord injury,^12^ Alzheimer’s disease,^13^ Parkinson’s disease,^14^ amyotrophic lateral sclerosis,^15^ and demyelinating diseases such as multiple sclerosis.^16^

Recent studies indicate that mitochondria can be released into the extracellular space and may be transferred between cells within the central nervous system. In injured or diseased brain tissue, these extracellular mitochondria and their components could act as a new form of intercellular signaling, with effects that may be either harmful or protective depending on the circumstances. Consequently, mitochondrial transplantation has emerged as a promising therapeutic strategy to restore function in damaged or diseased cells. The fast-growing research on mitochondrial transfer has underscored the importance of standardizing terminology within the field. In response, the International Committee on Mitochondria Transfer and Transplantation Nomenclature (ICMTTN) was recently formed to unify current knowledge on mitochondrial transfer mechanisms and therapies, as well as to offer recommendations on consistent terminology and characterization criteria.^17^ Following the published guidelines, we review key studies on endogenous mitochondrial transfer as a form of intercellular communication, examine the role of cell-free extracellular mitochondria and their components as emerging mediators and biomarkers, and ultimately consider the therapeutic potential and enhancements of mitochondrial transplantation in the treatment of stroke.

## Intercellular mitochondrial transfer in CNS pathophysiology

Mitochondria serve as the intracellular powerhouses essential for energy production and cell survival in the central nervous system (CNS).^18^ Maintaining mitochondrial quality control is, therefore, critical. Under normal conditions, mitochondria sustain their function through balanced cycles of fission and fusion. When mitochondria suffer excessive damage due to injury or disease, dysfunctional mitochondria fail to reintegrate into the network and are targeted for elimination via mitophagy—a selective autophagy process mediated by autophagosomes and lysosomes.^19^ The degradation process recycles amino acids and fatty acids, which are then reused for ATP generation,^20^^,^^21^ emphasizing mitophagy’s vital role in mitochondrial quality control and metabolic balance.

A comparable mechanism may operate within neuron-glial networks. For example, retinal neurons have been observed to transfer mitochondria to retinal astrocytes for disposal and recycling.^22^ Using serial block-face scanning electron microscopy, axonal protrusions from retinal ganglion cells containing mitochondria were visualized within astrocytes, where confocal microscopy confirmed their degradation. In Parkinson’s disease, this process, termed transmitophagy, appears crucial for preserving mitochondrial function in dopaminergic neurons and preventing the release of damaged mitochondria into the extracellular space, which could trigger neuroinflammation.^23^ Collectively, transmitophagy represents a form of mitochondrial quality control via intercellular transfer in the CNS.

Recent proof-of-concept studies have shown that astrocytes can also donate mitochondria to damaged neurons, promoting neuroprotection, and repair through CD38 signaling.^24–27^ Mutations in astrocytic GFAP disrupt this mitochondria transfer, accompanied by reduced CD38 expression,^28^ indicating CD38 as a potential target to modulate this process. A key question is how mitochondria maintain their functionality during release and transfer. Stimulation of astrocytic CD38 by nicotinamide adenine dinucleotide (NAD+) enhances O-GlcNAc post-translational modification of mitochondrial proteins, which supports neuroprotection after transfer.^29^ O-GlcNAcylation of mitochondrial proteins boosts energy production, membrane potential, and mitochondrial mobility,^30^ and dysregulation of this modification has been implicated in neurodegenerative diseases.^31–33^

The neuroprotective effect of astrocyte-derived mitochondria may depend on the astrocyte activation state. For instance, reactive astrocytes after stroke may express regenerative genes (A2), whereas neurodegenerative conditions induce a pro-inflammatory phenotype (A1).^34–38^ The A1 phenotype is associated with reduced mitochondrial protein O-GlcNAcylation.^39^ Additionally, microglia activated by LPS and A1 astrocytes have been shown to release fragmented, dysfunctional mitochondria into the extracellular space, which exacerbates neuronal death.^40^ This suggests that neuro-glial interactions mediated by dysfunctional extracellular mitochondria may contribute to the spread of neuronal injury in neurodegenerative disorders.

Beyond intrinsic CNS interactions, peripheral responses also appear to influence neurovascular remodeling.^41–43^ For example, human endothelial progenitor cells (hEPCs) can release functional extracellular mitochondria.^44^ Transfer of these mitochondria into brain endothelial cells enhanced viability and barrier integrity while restoring ATP and mitochondrial DNA levels following ischemic injury.^44^ Additionally, hematopoietic stem and progenitor cells (HSPCs) have been observed to transfer mitochondria to neurons, improving mitochondrial function and increasing frataxin expression in a mouse model of Friedreich’s ataxia.^45^ These findings lay a foundation for exploring mitochondrial transfer as a mode of communication between the damaged brain and peripheral tissues after stroke, brain injury, or neurodegeneration.

While the exact mechanisms underlying mitochondrial release and uptake are not yet fully understood, several pathways are currently under active investigation. One such mechanism involves tunneling nanotubes (TNTs), which are actin-based structures that form direct physical connections between donor and recipient cells, facilitating mitochondrial transfer. This process is thought to be regulated by proteins such as Miro1, along with cytoskeletal components and motor proteins like kinesin and myosin.^46^^,^^47^ Additionally, mitochondria or their fragments can be packaged into extracellular vesicles (EVs), including microvesicles and exosomes, with their transfer likely dependent on ESCRT complexes and integrin-mediated membrane trafficking.^24^^,^^48^ Recent research also suggests that interactions between mitochondria and the endoplasmic reticulum (ER) may aid mitochondrial transfer within osteocyte dendritic networks,^49^ highlighting the intricate nature of these intercellular communication pathways.^17^

## Role of mitochondrial dysfunction in worsening ischemic stroke

Mitochondrial dysfunction is increasingly recognized as a key factor in the development and worsening of ischemic stroke. After ischemia and subsequent reperfusion, damage to oxidative phosphorylation causes a decline in ATP production, disrupts ion balance, and leads to excitotoxic injury in neurons. Dysfunctional mitochondria contribute to cell death through necrosis in the ischemic core and promote apoptosis in the surrounding penumbra, exacerbating secondary injury.^50^ Moreover, excessive leakage of electrons from the mitochondrial respiratory chain generates high levels of reactive oxygen species (ROS), which harm cellular lipids, proteins, and DNA, thereby intensifying cell damage.^51^ Calcium overload triggers mitochondrial depolarization and the opening of the mitochondrial permeability transition pore (mPTP), releasing cytochrome c and activating apoptotic pathways that worsen ischemic injury.^50^^,^^52^

Disrupted mitochondrial dynamics further contribute to pathology: an imbalance characterized by increased mitochondrial fission (mediated by DRP1) and reduced fusion (due to lower levels of MFN1/2 and OPA1) leads to fragmented mitochondria with impaired energy production and greater susceptibility to cell death.^53^ Concurrently, ischemia inhibits mitophagy, preventing the removal of damaged mitochondria and allowing oxidative stress to accumulate.^53^ Additionally, the release of mitochondrial damage-associated molecular patterns (DAMPs), such as mitochondrial DNA (mtDNA) and cardiolipin, activates microglial cells and immune responses, amplifying neuroinflammation and tissue damage.^54^

Together, these mechanisms illustrate how mitochondrial dysfunction intensifies ischemic stroke pathology. This highlights dysfunctional mitochondria as both a central mediator of stroke damage and a promising target for therapeutic intervention.

## Cerebrospinal fluid mitochondria as potential biomarkers in stroke

Growing evidence indicates that respiratory-competent mitochondria and their components can be released into extracellular fluids such as blood^55^ and cerebrospinal fluid (CSF).^56^ These extracellular mitochondrial elements may actively participate in the pathophysiology of injury and disease,^57^ positioning them as a novel class of biomarkers. The underlying concept is that the functional state of extracellular mitochondria mirrors intracellular metabolic health; therefore, regardless of how they are released or reabsorbed, they can serve as indirect indicators of CNS metabolic integrity.^58^

Our research has shown that measuring mitochondrial membrane potential in CSF offers insights into mitochondrial function or integrity within the brain.^56^^,^^58^ Using the JC1 assay on human CSF samples, we observed a decrease in extracellular mitochondrial membrane potential following subarachnoid hemorrhage (SAH). Notably, patients exhibiting higher mitochondrial membrane potentials in CSF had better clinical recovery three months post-SAH.^56^ To identify the potential cellular origins of these CSF mitochondria in SAH, we examined extracellular mitochondria enclosed within membranous structures via transmission electron microscopy (TEM). By performing flow cytometry with markers such as GLAST (astrocytes), vWF (endothelial cells), CD45 (microglia/macrophages), and CD41/CD61 (platelets), we found that individuals with favorable outcomes at three months showed a significantly greater proportion of GLAST-positive mitochondria in their CSF three days after SAH, while no significant correlations were seen with other cell markers.

In another proof-of-concept study involving patients with aneurysmal SAH complicated by delayed cerebral ischemia (DCI), CSF samples were labeled with MitoTracker and cell-specific markers including GLAST, vWF, CD45, and CD41/CD61. Lower mitochondrial membrane potential and reduced GLAST-positive mitochondria, coupled with increased vWF-positive mitochondria in the CSF, were linked to DCI development, suggesting that extracellular mitochondrial function in CSF may predict future DCI risk.^59^

Mitochondrial components also appear to regulate immune and inflammatory responses.^60^ Cell-free mitochondrial DNA (mtDNA) contains damage-associated molecular patterns (DAMPs), such as CpG motifs, which can activate toll-like receptor 9 (TLR9), acting as danger signals.^60^^,^^61^ MtDNA can induce sepsis-like symptoms in systemic inflammatory response syndrome, resembling bacterial molecular patterns.^62^ Circulating mtDNA levels rise with age, especially after 50, correlating with chronic low-grade inflammation,^63^^,^^64^ indicating that mitochondrial DAMPs may contribute to age-associated inflammation. Extracellular mtDNA is detectable in CSF, and elevated mtDNA levels have been observed in various neurological conditions. For instance, Cervera-Carles et al. reported increased CSF mtDNA in Alzheimer’s disease patients compared to healthy controls,^65^ while Peng and colleagues found elevated mtDNA in the CSF of patients with anti-N-methyl-D-aspartate receptor encephalitis relative to controls.^66^

Emerging evidence suggests that extracellular mitochondria may serve as novel biomarkers in neurological diseases. Their presence in biofluids such as blood and cerebrospinal fluid reflects dynamic processes of cellular stress, injury, and intercellular communication. Quantitative and qualitative assessments such as mitochondrial DNA copy number, respiratory function, and membrane integrity have been associated with disease severity and outcomes in preclinical and clinical studies.^56^^,^^67^^,^^68^ While standardized methodologies for isolation and characterization are still being developed, the ability to monitor extracellular mitochondria in minimally invasive samples raises the possibility of using them as diagnostic or prognostic indicators, as well as pharmacodynamic markers to assess treatment response. Advances in high-resolution flow cytometry, single-organelle imaging, and next-generation sequencing may allow for more detailed characterization of extracellular mitochondria in clinical samples. Future studies are warranted to validate their specificity and sensitivity, and to determine whether distinct mitochondrial signatures can differentiate between disease subtypes or stages.

## Mitochondrial transplantation as an adjunct therapy for stroke

The maintenance of homeostasis in the central nervous system relies heavily on the intricate communication among neurons, glial cells, and vascular components—collectively referred to as the “neurovascular unit.“^69–74^ Disruption of mitochondrial signaling within this unit is increasingly recognized as a key factor underlying various CNS disorders. In a groundbreaking proof-of-concept study in cardiac tissue, McCully and colleagues demonstrated that autologous mitochondria isolated from a patient’s skeletal muscle could be directly transplanted into the heart without provoking immune or autoimmune reactions.^75^^,^^76^ This finding suggests that mitochondrial transplantation could potentially be adapted as a therapeutic strategy for other conditions, including CNS injuries such as stroke.

To date, over 400 clinical trials investigating mitochondrial-targeted interventions, including completed studies, have been registered at ClinicalTrials.gov. However, most have focused on small molecules or metabolic modulators, and therapies directly targeting mitochondrial function remain underdeveloped. Therapeutic interventions such as recombinant tissue plasminogen activator or endovascular thrombectomy effectively restore reperfusion in acute ischemic stroke (AIS).^50^ While restoring cerebral blood flow is essential to reducing infarction, reperfusion alone may be insufficient to salvage penumbral tissue in some patients. After ischemic stroke, deprivation of glucose and oxygen disrupts mitochondrial ATP synthesis, induces metabolic imbalance, impairs cellular homeostasis, and leads to neural cell death. Thus, targeting mitochondria represents a promising strategy for neuroprotection in stroke,^77–79^ and developing approaches to restore mitochondrial function to complement ischemia–reperfusion remains critical.^50^

Studies have shown that direct transplantation of healthy mitochondria can provide neuroprotection in animal models of focal cerebral ischemia. For example, in one study, mitochondria isolated from hamster kidney fibroblast (BHK-21) cells were injected either directly into the ischemic striatum or via intra-arterial administration after transient focal ischemia. To track the transplanted mitochondria, BHK-21 cells were pre-labeled with BrdU, which also marks mtDNA. Confocal microscopy detected BrdU signals in neurons, astrocytes, and microglia within the peri-infarct region up to four weeks post-ischemia, regardless of delivery method. Importantly, mitochondrial transplantation led to improved motor function, reduced infarct size, and fewer TUNEL-positive cells, indicating decreased apoptosis—supporting the potential effectiveness of xenogeneic mitochondria transplantation.^80^

Another study explored the use of mitochondria derived from mouse placenta in a focal cerebral ischemia model. Mitochondria isolated from snap-frozen placentae of E17 pregnant mice retained high viability, with up to 87% maintaining membrane potential as assessed by JC1 assay. These placental mitochondria contained ATP levels comparable to those from skeletal muscle and brown fat, and preserved antioxidant enzymes such as glutathione reductase, Mn-SOD, and HSP70. When administered intravenously immediately after reperfusion in a blinded, randomized setup, placental mitochondrial transplantation significantly reduced infarct size. This suggests cryopreserved placenta as a viable source for mitochondrial isolation with therapeutic potential to enhance reperfusion benefits in stroke.^81^

Additionally, Zhang et al. performed a long-term study transplanting skeletal muscle-derived mitochondria into the lateral ventricle of rats immediately after reperfusion in a transient focal ischemia model. Using Mitotracker Red labeling, they confirmed incorporation of transplanted mitochondria into peri-infarct neurons. Mitochondrial transplantation reduced infarct volume, mitigated neurological deficits, and decreased oxidative stress and apoptosis up to 28 days poststroke. Furthermore, treatment reduced reactive astrogliosis and promoted neurogenesis, indicating that mitochondrial transplantation may offer both acute neuroprotection and support recovery in later stages of stroke.^82^

Although mitochondria transplantation has emerged as a promising approach, major limitations remain. First, a common dosing unit such as protein content (µg), number of mitochondria, or mitochondrial activity has yet to be established. Converting between these units is challenging, for example, from protein concentration to mitochondrial number, due to variability in the quality and purity of isolated mitochondria. Therefore, it would be preferable to assess and report at least two parameters including protein concentration, mitochondrial number, or mitochondria-specific enzyme activity alongside experimental results. Second, a standardized method to evaluate mitochondrial quality and activity remains lacking. Potential candidates include membrane potential measurement, TEM analysis, and citrate synthase activity. However, methods that are simple, robust, and widely accessible to laboratories would be preferable. Additionally, the optimal source of mitochondria (autologous vs allogenic) and the most effective formulation (fresh vs freeze-thawed) for restoring injured tissue have yet to be fully determined. Future discussions and studies are warranted to establish clear guidelines addressing these critical issues.

## Next generation of mitochondrial transplantation therapy

Despite growing evidence supporting the therapeutic potential of mitochondrial transfer, several challenges remain. Mitochondria are highly vulnerable in extracellular environments, and the efficiency of their uptake by recipient cells can be limited. Additionally, certain harmful modifications on mitochondria or their surfaces—such as oxidized cardiolipin and advanced glycation end products—may reduce their therapeutic efficacy or even cause adverse effects.^83^^,^^84^ In light of these challenges, we outline three innovative strategies for mitochondrial transplantation therapy, each inspired by endogenous mechanisms observed within the CNS.

### O-GlcNAcylation

Park and colleagues have shown the efficiency of O-GlcNAcylation of mitochondria to support extracellular mitochondria functionally.^29^^,^^84^ In this study, they found the activation of CD38-cADPR signaling in the astrocytes significantly increased protein O-GlcNAcylation in mitochondria, while oxygen-glucose deprivation (OGD) and reoxygenation showed transient and mild protein modification. Interestingly, O-GlcNAcylated mitochondria demonstrated higher membrane potential, mtDNA content, and neuroprotective capacity when transferred to neurons damaged by OGD. Furthermore, when O-GlcNAcylation was blocked by Brefeldin A, these effects were significantly decreased. In addition to these findings of *in vitro* experiments, the same group found that mitochondrial modification with O-GlcNAc by UDP-GlcNAc and protein O-GlcNAc transferase protected mitochondria against glycation and enhance mitochondrial viability in the extracellular environment *in vitro*. They also found O-GlcNAc modified mitochondria reduced infarct volume and improved neurologic deficits in a middle cerebral artery occlusion (MCAo) model mouse. Although *ex vivo* chemical modification may alter the regulatory classification of the product and require different testing, these results suggest that O-GlcNAc modification of mitochondria is a promising strategy to maintain mitochondrial viability and quality prior to mitochondria transplantation therapy.

### Mitochondrial surface coating

Nakano et al. developed encapsulation method of mitochondria within artificial lipid membrane being hinted from that astrocytic mitochondria were appeared to be encapsulated within extracellular vesicles.^24^^,^^85^ They attempted to encapsulate mitochondria with cationic and fusogenic lipids, 1,2-dioleoyl-*sn*-glycero-3-phosphoethanolamine (DOPE) and 1,2-dioleoyl-3-trimethylammonium-propane (DOTAP)^86^^,^^87^ to protect mitochondria from extracellular environment and improve mitochondria transfer and efficiency. FACS analysis confirmed that around 90% of mitochondria can be covered by DOPE/DOTAP membrane successfully, and mitochondrial proteins and membrane potentials were maintained without any significant compromises. Using DOPE/DOTAP covered mitochondria, they tested the efficiency of mitochondria transfer and neuroprotection. The number of DOPE/DOTAP covered mitochondria in damaged neurons, which were mouse primary and examined oxygen-glucose depression (OGD) treatment, was higher than that of noncovered mitochondria. Furthermore, compared with noncovered mitochondria, treatment of DOPE/DOTAP covered mitochondria can be improved damaged neurons viability at 24 h after OGD. Moreover, compared to uncoated mitochondria, intravenous infusion of DOPE/DOTAP-coated mitochondria immediately following focal cerebral ischemia–reperfusion significantly increased the accumulation of infused mitochondria within the ischemic region and enhanced cerebroprotection.^85^ While hemocompatibility and complement activation testing may be necessary when systemic delivery is considered—given that cationic lipids can influence vascular compatibility in clinical settings—DOPE/DOTAP encapsulation represents a promising modification that could improve the therapeutic efficacy of mitochondrial transfer. Importantly, because the blood–brain barrier (BBB) remains permeable for days to weeks following ischemic stroke,^88^ and our previous studies have demonstrated that mitochondrial coating enhances delivery of mitochondria to injured brain tissue after intravenous infusion poststroke,^85^ the DOPE/DOTAP encapsulation strategy may hold particular translational relevance across the acute to subacute phases of stroke therapy.

### MitoEVs

Recently, extracellular vesicle (EV)-encapsulated mitochondria have been reviewed for their potential in treating ischemic stroke. Cell-derived EVs naturally contain mitochondrial components, which can protect their contents from degradation.^89^ Importantly, EVs can cross the BBB to deliver cargo to target cells in the central nervous system,^90^^,^^91^ highlighting their promise as carriers for brain-targeted therapies.^92^^,^^93^ It has been reported that muscle tissue–derived MitoEVs, when administered allogenically to injured muscle, can restore mtDNA levels, enhance mitochondrial biogenesis, and improve mitochondrial function.^94^ Notably, Dave and colleagues compared mitochondria transfer efficiency of EVs in same-species versus cross-species settings.^95^ They found that EVs derived from mouse brain endothelial cells (mBEC-EVs) significantly outperformed EVs from human brain endothelial cells (hBEC-EVs) in enhancing ATP production and oxygen consumption in recipient mBECs *in vitro*. Furthermore, mBEC-EVs markedly reduced brain infarct volume and improved neurological outcomes in a mouse model of middle cerebral artery occlusion (MCAo). These findings suggest that allogenic mitochondria-containing EVs may offer superior therapeutic efficacy, providing a more translationally relevant strategy for future preclinical and clinical development.

## Clinical implication of mitochondria transplantation in stroke

Excitingly, mitochondria transplantation therapy may be clinically applicable in stroke. Walker et al. have reported the results of Phase I trial of autologous mitochondrial transplantation for the treatment of AIS in human patients.^96^ In the published first-in-human brain study, autologous mitochondria were isolated from skeletal muscle and delivered intra-arterially into the middle cerebral artery during thrombectomy. Safety endpoints included intraprocedural complications such as hemorrhage or thrombus formation, postprocedural hemorrhage, systemic adverse events within the first week, and biopsy-site complications. No significant adverse events were observed during the endovascular procedure, and safety outcomes were comparable to those in matched control groups without mitochondrial transplantation. The authors highlighted several critical considerations for broader clinical translation, including the localization and functional verification of transplanted mitochondria within the brain, the optimal timing and frequency of transplantation, and the most effective delivery route. Moreover, Walker et al. recently established a radiolabeling method to track administered mitochondria. Cryopreserved mitochondria were labeled with technetium-99m (Tc-99m) without compromising ATP production or cristae structure, and this platform supports the development of a SPECT-compatible protocol for monitoring viable transplanted mitochondria in recipient tissues.^97^ Although the half-life of Tc-99m is approximately 6 h, limiting long-term visualization, this advance represents an important step toward overcoming a key translational barrier.

In addition to above research, mitochondrial modification may offer a potential solution to these challenges. For example, O-GlcNAcylation has been shown to enhance mitochondrial viability in the extracellular environment *in vitro*, and to reduce infarct volume and improve neurological outcomes in ischemic stroke model mice. Furthermore, modifications such as DOPE/DOTAP coating and extracellular vesicle encapsulation have demonstrated therapeutic benefits in both *in vitro* and *in vivo* experiments. Collectively, these strategies suggest that mitochondrial modification represents a promising therapeutic approach for AIS and other central nervous system injuries, with potential applications extending from preclinical research to future clinical translation.

While progress has been made, acute stroke workflows leave little room for additional steps during thrombectomy, whereas subacute settings allow more flexibility through intravenous delivery. Clarifying these operational constraints is essential to align manufacturing strategies with clinical feasibility, patient selection, and trial design.

## Xenogeneic and allogeneic mitochondria transplantation

Xenogeneic and allogeneic mitochondrial transplantation present distinct opportunities and challenges for both basic research and clinical translation. Xenogeneic transplantation, involving the transfer of mitochondria between different species, serves primarily as a research tool to explore mitochondrial compatibility, immune recognition, and intercellular organelle transfer. Nakamura and colleagues investigated the dose-dependent uptake and molecular effects of xenogeneic mitochondrial transplantation using rat-derived mitochondria in mouse neuronal systems. Their results demonstrated successful mitochondrial uptake and integration, supporting the feasibility of transfer across species barriers, although the persistence, localization, and functional contribution of the transferred mitochondria remain to be fully elucidated.^98^ Xenogeneic mitochondrial treatment has also been tested during *ex vivo* lung perfusion to mitigate ischemia-reperfusion injury, with findings indicating improved lung function and no acute rejection of mitochondria.^99^ Collectively, these studies highlight the potential of xenogeneic mitochondrial transplantation as a therapeutic strategy across diverse tissues and conditions. While feasibility has been demonstrated, further research is required to clarify underlying mechanisms and optimize protocols for future clinical application.

Conversely, allogeneic mitochondria transplantation involves transferring mitochondria between genetically distinct individuals of the same species, aiming to restore mitochondrial function in damaged or diseased cells. This approach offers the advantage of providing healthy mitochondria without the need for invasive harvesting from the patient, enabling more rapid therapeutic intervention. Importantly, although allogeneic mitochondria differ genetically from the recipient’s own mitochondria, there is currently no direct experimental evidence of immune rejection specifically triggered by healthy mitochondria, although immune responses remain a theoretical concern and require further investigation. This uncertainty underscores the importance of more research to define the safety profile of allogeneic mitochondrial transplantation, including long-term integration and functional persistence in recipient cells.

From this perspective, xenogeneic and allogeneic transplantation offer complementary strategies for advancing mitochondrial therapy, although their clinical translation demands careful preclinical validation, immune compatibility assessment, and ethical consideration to ensure safety and efficacy.

## Conclusions

Mitochondrial integrity plays a crucial role in CNS recovery following injury or disease.^18^^,^^100^ Recent studies across cellular, animal, and human models reveal that mitochondria can be released into the extracellular space and transferred between cells. Moreover, extracellular mitochondria are detectable in human samples, with their functional status correlating with the severity of injury or disease. This highlights their potential as a novel class of signaling mediators and biomarkers. Within the evolving framework of mitochondria-targeted therapies for CNS disorders,^101–103^ the therapeutic application of extracellular mitochondria emerges as a promising avenue for treating a broad spectrum of neurological conditions. Additionally, recent animal research suggests that modifying mitochondria can improve their stability and enhance neuroprotective effects. Notably, the establishment of the ICMTTN has standardized terminology to minimize confusion in the field.^17^ This global scientific network fosters collaboration among researchers worldwide, encouraging the exchange of ideas and accelerating the development of safer, more effective mitochondrial therapies—from basic science to clinical application—across various mitochondria-related diseases.

## Data Availability

No new data were generated or analyzed in support of this research.
